# Notes on Preparations for the Sick

**Published:** 1907-03

**Authors:** 


### Notes on Preparations for the Sick.

---

**Colalin; Colalin Laxative.**—T. MORSON & SON, London.—These two preparations are in tablet form, the dose of colalin being half a grain, one tablet to be taken three or four times a day. It is considered to be pure cholalic acid, an amorphous powder, insoluble in the stomach, but soluble in the upper intestines, from whence it is absorbed, as no traces of cholalin are found in the fæces. It is believed to stimulate the liver and cause an increased flow of bile.

The laxative tablet contains a combination of cholalin with the anthraquinone principle of cascara. These bile preparations are said to be of genuine therapeutic value in cases of functional affections of the liver; the promoters of them state that they may be absolutely relied upon to stimulate the flow of bile, and more especially to convert an abnormal viscous bile into a normal and viscid one. In one case the effect of this tablet on the bowels was found to be insignificant.

---

**Liquid Somatose.**—BAYER CO. LIMITED, 19 St. Dunstan's Hill, London, E.C.—This preparation is introduced for the convenience of those who find the powdered somatose troublesome to dissolve. The nutrient properties are mainly due to the presence of proteoses and alkali albumen; the unsweetened solution gives no precipitate on boiling, but a dense precipitate on addition of an acid, which completely disappears on heating, and reappears on cooling. The biuret reaction gives a pink colour, with dilute solutions. There are two varieties of Liquid Somatose, a sweetened and unsweetened, the latter being available for diabetics, and the former recommended for children. Both are quite palatable, and the former may be added to soup or beef-tea without spoiling the flavour of these dishes.

---

**Formamint Tablets.**—A. WULFING & CO, 83 Upper Thames Street, London.—Formamint is a chemical combination of formic aldehyde with lactose. The tablets dissolve readily in the mouth ; they produce an abundant flow of saliva, which, containing formic aldehyde, has an effective antiseptic action in the throat. They are considered to be the best substitute for gargles, and as they are to be dissolved slowly in the mouth, their action is likely to be much more prolonged. They are quite palatable and non-irritating, so that they may be readily taken by children, and should be effective in all cases of sore throat when a non-irritating antiseptic is desirable.

---

**Tabloids : Guaiacol Camphorate,** gr. v. ; **Calcium Lactate,** gr. v. ; **Pectoral Pastilles.**—BURROUGHS, WELLCOME & OO., London.—The Guaiacol Camphorate Tabloid should be a convenient form of administration of two useful drugs in cases of phthisis. The guaiacol has deservedly established a reputation, and in its combination with another drug also of established reputation, camphoric acid, its value should be greatly enhanced.

Calcium Lactate is employed to increase blood coagulability, which has been shown to be deficient in cases of urticaria and other affections. Its administration has proved successful in the treatment of urticaria, chilblains, certain forms of albuminuria, headache, and serum rashes. It is also employed in aneurism and in various forms of hemorrhage, including the hemorrhagic form of small-pox ; as a preventive and curative in hæmophilia, in uterine hemorrhages, and preliminary to surgical procedure. "Tabloid" Calcium Lactate presents a convenient and satisfactory means of administering a calcium salt. Calcium Lactate is now preferred to the chloride, which has sometimes been found to produce disturbance of the alimentary canal. This tabloid is non-irritating, readily soluble and easily absorbed. One or more may be taken twice or thrice daily for two to three days.

"Tabloid" Pectoral Pastilles contain ammoniated liquorice, squill, tolu, senega, ipecacuanha, wild cherry, etc. They afford a palatable and convenient means of exhibiting aromatic, expectorant, demulcent and sedative principles. Slowly dissolved in the mouth, these pastilles exert a prolonged and uniform effect on the respiratory tract. They relieve cough, check excessive secretion, and soothe the irritated mucous membrane. They should be valuable in colds, hoarseness, and bronchial affections.

---

**Phenofax.**—BURROUGHS, WELLCOME & Co., London.—This antiseptic surgical dressing contains 7 per cent. of pure phenol in a bland basis. It may be applied on lint, or may be used as an ointment in parasitic skin diseases. It is also suitable for application to affected mucous surfaces, more especially ulceration of the cervix uteri.

**Soloid—Eosin-azur.**—BURROUGHS, WELLCOME & Co., London. —We have examined this "Soloid." Each soloid contains 0.015 gm. of Giemsa's stain, and is dissolved in 5 c.c. of pure methyl alcohol. When the staining directions are properly followed, the results are thoroughly satisfactory. These "soloid" stains are most convenient for use, and are reliable as regards their staining properties.

